# Stepping-forward affordance perception test cut-offs: Red-flags to identify community-dwelling older adults at high risk of falling and of recurrent falling

**DOI:** 10.1371/journal.pone.0239837

**Published:** 2020-10-08

**Authors:** Catarina Pereira, Jorge Bravo, Guida Veiga, José Marmeleira, Felismina Mendes, Gabriela Almeida

**Affiliations:** 1 Departamento de Desporto e Saúde, Escola de Ciências e Tecnologia, Universidade de Évora, Évora, Portugal; 2 Comprehensive Health Research Centre, Lisboa, Portugal; 3 Escola Superior de Enfermagem S. João de Deus, Universidade de Évora, Évora, Portugal; West Virginia University, UNITED STATES

## Abstract

The stepping-forward *affordance* perception test (SF-APT) fills an important gap within the screening of falls risk factors by considering the perception of *affordances*. The test showed to be a valid instrument for community-dwelling older adults falls risk assessment. The present study aimed to distinguish and test the key outcomes of the SF-APT usable for falls risk assessment in community-dwelling older adults to determine the respective cut-offs. This cross-sectional study enrolled 347 participants (73.1 ± 6.2 years; non-fallers: 57.9%; fallers: 42.1%; recurrent-fallers: 17.9%). Falls occurrence and SF-APT outcomes were assessed. Analyses were performed using multivariate binary logistic regression analysis and receiver operating characteristic (ROC). The area under the ROC curve was computed (AUC) for each built model explaining falling or recurrent falling. Results distinguished the Estimated stepping-forward, and Absolute-error in interaction with Error-tendency as the SF-APT key outcomes for falls risk assessment [AUC_falling_: 0.665 (CI 95%: 0.608–0.723); AUC_falling recurrently_: 0.728 (CI 95%: 0.655–0.797)]. Computed cut-offs’ analysis showed that (i) a boundary stepping-forward estimation >58 cm plus an underestimation bias >5 cm (>42^nd^ percentile) avoid older adults to be recurrent-fallers, and (ii) a boundary stepping-forward estimation >62 cm plus an underestimation bias >6 cm (>54^th^ percentile) avoid older adults to be fallers. In conclusion, results suggest that SF-APT is a valuable tool for falls risk assessment in community-dwelling older adults. Interventions targeting the prevention of falls should consider the above key outcomes and the respective cut-offs as alert red-flags.

## Introduction

Researchers have been looking for tools to identify older adults at high risk for falls [1–3], mainly because falls risk assessment represents a crucial strategy to prevent falls occurrence [4]. Recently, Palumbo et al. [5] computed an algorithm explaining falls occurrence, which included previous falls, gait, and balance. Nonetheless, the ability of previously mentioned falls risk assessment tools has shown a low-moderate ability to correctly discriminate those older adults who fall, suggesting a gap in the assessed outcome used for falls risk screening. An ecological framework was developed by Gibson as regards the concept of *affordance* concerning the relationship between perception and action [6,7]. According to *affordance* theory, opportunities for actions emerge under a particular set of conditions and body characteristics [8]. A safe and successful action performance would depend on personal self-perceived action boundaries, and a perceptual misestimation in locomotor skills in older adults may lead to an accidental fall [9]. Luyat et al. [10], have already reported that ageing-associated misperception of *affordances* could be a risk factor for falling in older adults. Thus, we hypothesized that the *affordance*´s perception could be a key element for being considered on falls risk assessment.

A recent study developed and examined the validity and the reliability of the Stepping-Forward *Affordance* Perception Test (SF-APT), which has shown to be a valid instrument for community-dwelling older adult’s falls risk assessment [11]. This test measures the perceived stepping-forward boundary as well as the real stepping-forward boundary, such that the judgment bias (real performance–estimation) provides information about actual performance and the perceived action capability. The SF-APT is a valid instrument for community-dwelling older adults falls risk assessment [11], which fills an important gap within the screening of falls risk factors [2,3,12] by considering the perception of *affordances* [11]. Besides, this test is quick and easy to administer and almost inexpensive. Several authors have provided cut-offs values of risk for falls concerning their measuring tools, such as for the Berg Balance Scale [13], the Fullerton Advanced Balance Scale [14], or the Performance Oriented Mobility Assessment [15]. Such cut-off values work as red-flags of alert, which may be compared to the patient tests performance, allowing health professionals, family members, and patients themselves to become aware of their falls risk status [16,17]. However, the role of SF-APT outcomes on falls occurrence is still unclear; particularly, the cut-off values which best discriminate non-fallers from fallers and recurrent-fallers are unknown. Hence, the establishment of SF-APT key outcomes for falls risk assessment and the determination of the respective cut-off values of risk will allow researchers and clinicians to quantify the remoteness between individual SF-APT scores and the recommended reference values in each key outcome. Therefore, it will be possible to interpret the SF-APT results and to identify the necessary changes (which and how much) to avoid the threat of falls. This approach may be useful to the design of more effective falls’ prevention programs.

Thus, the present study aims to distinguish the SF-APT outcomes, which should be used for falls risk assessment in community-dwelling older adults, and determine the respective cut-offs usable to identify people at high risk of falling and of falling recurrently.

## Material and methods

### Participants

Three hundred and sixty-seven Portuguese community-dwelling older adults were originally enrolled in the study. The participants were recruited by invitation and through printed flyers distributed in community settings (including sports*/*recreation centers). The inclusion criteria were age ≥65 years old, living independently in the community, absence of falls occurrence due to hazardous occurrences and unusual tasks, and absence of cognitive impairment according to the Portuguese version of the Mini-Mental State Examination [18,19]. Twenty volunteers were excluded: 11 showed cognitive impairment, and 9 had fallen due to hazardous occurrences and unusual tasks, such as jumping over a wall with a bicycle, going rollerblading, or, unusually, acting as a longshoreman to change the furniture in the house. Thus, 347 older adults (aged 73.1 ± 6.2 years; with 5.2 ± 3.7 years of school; non-fallers: 57.9% *vs*. fallers: 42.1%; recurrent-fallers: 17.9%) were included in the study. Of them, 76.7% were female (height: 152.6 ± 6.4 cm, BMI: 29.0 ± 4.0 m/kg^2^, and body fat mass percentage 41.0 ± 6.4%) and 23.3% were male (height: 166.6 ± 6.8 cm, BMI: 28.2 ± 3.7 m/kg^2^, and body fat mass percentage: 26.4 ± 6.2%). Ethical approval was obtained from the Ethics committee for research in the areas of human health and well-being–Universidade de Évora (reference number 16–012), and informed written consent was provided by the participants.

### Data collection

Participants were assessed individually for all measures by a multidisciplinary team, including human kinetics, rehabilitation, nursing, and gerontology fields, who attended a course on the measuring protocols. The evaluators were blind to the study’s objectives. Intra-rater reliability (intraclass correlation coefficient (ICC) ranging from 0.83 to 0.97) and in inter-rater reliability (ICC ranging from 0.99 to 1.00) showed a good test-retest reliability.

#### Perception and stepping-forward boundaries

The SF-APT was used to assess the perception and stepping-forward boundaries, and its protocol is described in detail elsewhere [11]. In order to avoid learning bias, a scoring attempt was preceded by a trial attempt, without any feedback between attempts. The following main outcomes are measured: Estimated stepping-forward (cm) and Real stepping-forward (cm). Posterior computation was performed to produce the following outcomes: Algebraic-error (Real performance–Estimation), Absolute-error (|Algebraic-error|), Absolute-percent-error (|1 –Estimation/Real performance| x 100) and Error-tendency which concerns the error direction (overestimation: Real<Estimated *vs*. underestimation: Real>Estimated) [20]. These computed variables measure the error between the real action boundary and the precepted action boundary, which is the judgment bias magnitude and respective direction.

#### Falls

The occurrence of falls in the previous 12 months and the circumstances surrounding each fall (such as where did the fall occur, what action did it take, what was the reason for the fall, which were the fall-related injuries) were carefully assessed by an interviewer who filled out a questionnaire. A non-faller was defined as a person who had not fallen in the previous 12 months, a faller as a person who had fallen at least once in this period, and a recurrent faller as a person who had fallen more than once in the same period [16,21].

#### Complementary measures

Sociodemographic characteristics were assessed by a questionnaire filled out by the interviewer. Height and weight were evaluated by using a stadiometer (Secca 770, Hamburg, Germany) and an electronic scale (Secca Bella 840, Hamburg, Germany), to compute body mass index (m/kg^2^). Body fat and lean mass were measured [22] by bioimpedance (Omron BF 511, USA).

### Data analysis

Descriptive statistics, based on mean and 95% confidence interval (CI), relative frequencies, and percentiles, were used to characterize the SF-APT outcomes participants’ data and illustrate fall occurrence. An exploratory analysis of the relationship between Estimated and Real stepping-forward outcomes was performed using Pearson’s correlation test.

In a previous study [11], multivariate binary logistic regression analysis and receiver operating characteristic (ROC) analysis were used to identify the key variables (outcomes) from the SF-APT, which should be included in the falls risk assessment. In the present study, these same techniques were used to test alternative models to the ones found in the previous research of Almeida et al. [11]—and respective key variables—as well as to quantify the role of each selected key variable on falls occurrence. For that, an overestimation error-tendency was coded as 0, and an underestimation error-tendency was coded as 1. Therefore, the internal validation of both most parsimonious and fit models (fallers *vs*. non-fallers and recurrent-fallers *vs*. non-fallers) was tested by using a resampling or cross-validation procedure [23]. For cross-validation, participants were clustered into ten equal groups by sampling randomly without replacement. The area under the ROC curve (AUC) was calculated using the probabilities that were generated by cross-validation [24]. Finally, multivariate binary logistic regression analysis and ROC analysis were used again to establish the cut-offs of risk of falling and of recurrent-falling. The cut-off point for falling probability was established by maximizing both sensitivity and specificity of the built and validated model. Therefore, the outlined cut-off point for falling probability was used to compute the percentile and correspondent absolute value on each SF-APT key variable, which best discriminates fallers from non-fallers. For this, the equation generated by regression modeling was successively solved using the respective key variables absolute values from the 1^st^ to the 99^th^ percentile. The percentile and correspondent absolute values that equaled the mentioned above falling probability cut-off point were identified as the cut-off values for each key variable (point estimation); namely, the cut-off values usable to discriminate fallers from non-fallers were identified. The same procedure was used to establish the cut-offs regarding falling recurrently. A similar methodology has been previously carried out [24].

Statistical analyses were performed using the SPSS package version 24 (SPSS Inc, Chicago, IL, USA). Statistical significance was set as *p* < 0.05.

## Results

Participants’ results on the SF-APT variables according to falls occurrence are displayed in Table 1. Descriptive analysis shows that in general, either the Estimated stepping-forward or the Real stepping-forward were smaller for fallers than for non-fallers and even smaller for recurrent-fallers. This analysis concerning error results evidenced that, in general, the bias between Estimated and Real stepping-forward was smaller in fallers than in non-fallers, and that this bias was even smaller in recurrent-fallers. Moreover, it was observed that the percentage of non-fallers showing an underestimation Error-tendency bias was higher than the percentage of fallers and recurrent-fallers showing this same Error-tendency. Hence, the underestimation bias was more prevalent for all participants (either non-fallers, fallers, or recurrent-fallers). However, the overestimation bias was more frequent in recurrent-fallers than in fallers, and more frequent in fallers than in non-fallers. Also, it should be noted that the analysis of the percentile values denotes a great variation of the SF-APT outcomes data. Finally, correlation analysis evidenced a positive relationship between Estimated and Real stepping-forward (r = 0.847, *p* < 0.001).

**Table 1:** Description of participants SF-APT outcomes data.

	Mean (95%CI) or prevalence				P5	P10	P25	P50	P75	P90	P95
	Non-fallers	Fallers	Recurrent-fallers	Total							
Estimated stepping-forward (cm)	63.7 (61.6–65.9)	57.1 (54.7–59.5)	55.4 (52.0–58.7)	60.9 (59.3–62.6)	36	41	50	61	71	80	88
Real stepping-forward (cm)	70.7 (68.6–72.7)	61.7 (59.4–64.1)	58.6 (55.3–61.9)	66.9 (65.3–68.5)	41	45	58	67	76	85	94
Algebraic-error^a^ (cm)	7.0 (5.7–8.2)	4.6 (3.4–5.9)	3.3 (1.3–5.3)	6.0 (5.1–6.9)	-6	-3	1	5	10	18	22
Absolute-error (cm)	8.4 (7.4–9.5)	6.7 (5.7–7.7)	6.5 (5.2–7.9)	7.7 (7.0–8.4)	0	1	2	6	11	18	22
Absolute-percent-error (%)	11.9 (10.4–13.3)	11.0 (9.5–12.5)	11.3 (9.0–13.5)	11.5 (10.5–12.5)	0.4	1.5	3.3	9.1	16.7	25.8	33.3
Error-tendency (%)											
Overestimation	17.9	29.5	33.9	22.8		--	--	--	--	--	--
Underestimation	82.1	70.5	66.1	77.2		--	--	--	--	--	--

^a^Real-Estimated.

Data are Mean and 95% Confidence Interval (CI) or Prevalence. and Percentile Values (P).

Table 2 presents the results of the two most parsimonious and fit models, built by multivariate binary regression, explaining falling and falling recurrently regarding the SF-APT outcomes. This Table illustrates that the multivariate binary regression analysis selected the variables Estimated stepping-forward and Absolute-error in interaction with Error-tendency as the key variables from the SF-APT, which should be included in falls risk assessment. These variables selection was computed both for assessing the risk of falling and for assessing the risk of falling recurrently, *p* < 0.05. Age and gender were not selected as significant factors explaining fall occurrence in multivariate modeling. Hence, in both models described above it was observed that, the larger the Estimated stepping-forward was, the lower the likelihood of falling (~3.6% for each additional cm) and of falling recurrently (~4.9% for each additional cm); and that, the larger the Absolute-error was, if the Error-tendency was of underestimation, the lower the likelihood of falling (5.9% for each additional cm) and of falling recurrently (8.6% for each additional cm). The Hosmer-Lemeshow goodness-of-fit test was not significant either regarding the falling model (*p* = 0.591) or the falling recurrently model (*p* = 0.241). Regarding the falling model, a sensitivity of 67.1% and a specificity of 58.7% were observed for the computed optimal cut-off point of 0.412 (41.2%) for the probability of falling. This model revealed an AUC of 0.665 (CI 95%: 0.608–0.723), and the predicted probabilities that were generated by the cross-validation procedure showed an AUC of 0.587 (CI 95%: 0.527–0.647). In what concerns to the falling recurrently model, a sensitivity of 66.1% and a specificity of 71.6%, were observed for the computed optimal cut-off point of 0.261 (26.1%) for the probability of falling recurrently. This model revealed an AUC of 0.728 (CI 95%: 0.655–0.797), and the predicted probabilities that were generated by the cross-validation procedure showed an AUC of 0.605 (CI 95%: 0.525–0.685).

**Table 2:** The two most parsimonious and fit build binary logistic regression models and respective key SF-APT variables explaining falling and falling recurrently (falling vs. non-falling model: N = 347; falling recurrently vs. non-falling model: N = 263).

Model	Key variables	OR (95%CI)	Optimal Cut-off point	Specificity (%)	Sensitivity (%)	AUC (95%CI)
Falling	Estimated stepping-forward (cm)	0.964 (0.948–0.979)	0.412	0.587	0.671	0.665 (0.608–0.723)
	Absolute-error (cm) * Error-tendency ^a^					
	Overestimation (0)					
	Underestimation (1)	0.941 (0.910–0.973)				
Falling recurrently	Estimated stepping-forward (cm)	0.951 (0.931–0.973)	0.261	0.716	0.661	0.728 (0.655–0.797)
	Absolute-error (cm) * Error-tendency					
	Overestimation (0)					
	Underestimation (1)	0.914 (0.868–0.962)				

*Interaction between variables.

Data are Multivariate Odds Ratio (OR) and 95% Confidence Interval (CI), Cut-off points for π, Specificity, Sensibility, and Area Under the ROC Curve (AUC) and 95% CI.

It is important to note that, in the modeling procedure, two similar multivariate binary regression models were built (with the same variables included in the models explaining falling and falling recurrently, as shown in Table 2). In both models, the reference category for the variable Error-tendency was “underestimation bias”. In this analysis, it was observed that, when an overestimation Error-tendency bias occurred, for each additional cm on the Absolute-error, there was an increase of 8.9% of the likelihood of falling [OR: 1.089 (95% CI: 1.001–1.185)] and there was an increase of 10.8% of the likelihood of falling recurrently [OR: 1.108 (95% CI: 1.043–1.177)].

**Fig 1:**
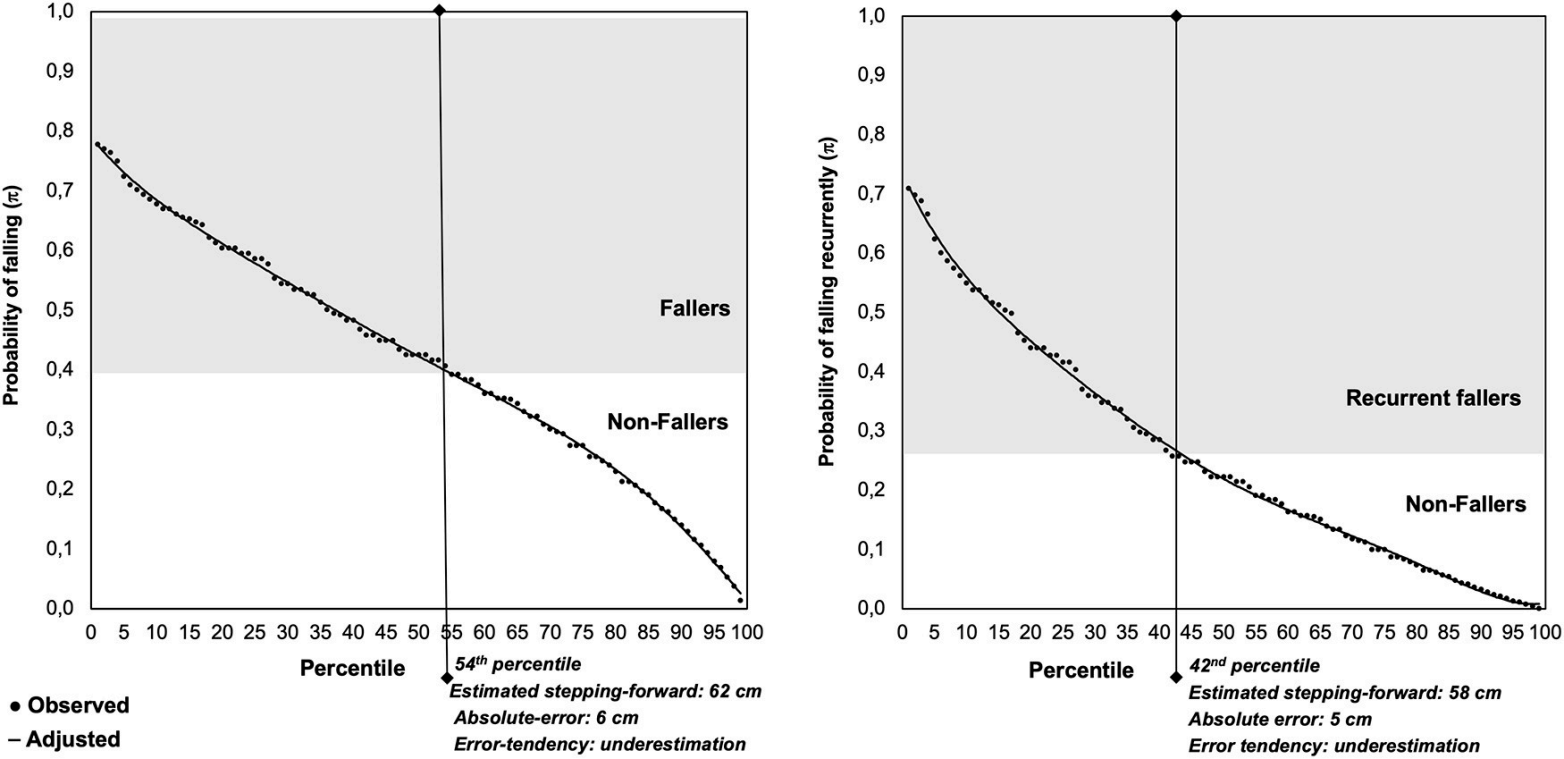
Probability falling and of falling recurrently associated with the key SF-APT outcomes (estimated stepping-forward, and the Absolute-error in interaction with Error-tendency) and respective cut-offs discriminating fallers from non-fallers or recurrent-fallers from non-fallers.

Fig 1 illustrates the change of the probability (point estimation) of falling and of falling recurrently as a function of Estimated stepping-forward and Absolute-error in interaction with Error-tendency (underestimation). A decrease in the likelihood of falling and in the probability of recurrently failing was observed with the increase in the Estimated stepping-forward, and the increase in the underestimated Absolute-error. Regarding the cut-off point (0.412), fallers were identified as those who estimated a boundary stepping-forward smaller than 62 cm and who showed an underestimated Absolute-error shorter than 6 cm. These cut-offs values correspond to the 54^th^ percentile. Regarding the cut-off point (0.261), recurrent-fallers were identified as those who estimated a boundary stepping-forward smaller than 58 cm and who showed an underestimated Absolute-error shorter than 5 cm. These cut-offs values correspond to the 42^nd^ percentile.

## Discussion

The results from the present study suggest that *affordance*´s perception works as a key component of falls risk assessment. Above all, this study results validated and confirmed (through a cross-validation procedure) that the best models to explain either falling recurrently or falling include the Estimated stepping-forward and the Absolute-error in interaction with Error-tendency as key SF-APT outcomes). Both models showed that there was a quantitative and a qualitative relationship between the participants’ results on these variables and the risk of falls; that is, the larger the Estimated stepping-forward was, the lower the likelihood of falling recurrently and of falling; and, in cases of Error-tendency underestimation bias, the larger the Absolute-error was, the lower the likelihood of falling recurrently and of falling. Moreover, data analysis identified cut-off values as red-flags of alert for the risk of falling recurrently and falling, which discriminate non-fallers from recurrent-fallers and non-fallers from fallers, corresponding to the 42^nd^ and 54^th^ percentiles, respectively. Our results suggest that to avoid being a recurrent-faller, older adults should estimate a boundary stepping-forward higher than 58 cm and underestimate an Absolute-error larger than 5 cm (i.e., above the 42^nd^ percentile). Besides, to avoid being a faller, older adults should estimate a boundary stepping-forward higher than 62 cm and underestimate an Absolute-error larger than 6 cm (i.e., above the 54^th^ percentile). These values were valid for both genders, independently of age.

Regarding the SF-APT outcomes, the results of the present study are consistent with the studies focused on community-dwelling older adults’ risk of falls, which found that poor performance in physical abilities is associated with an increased risk of falls [14,25,26], particularly if involving gait or stepping ability [27]. The present study showed that for non-fallers, the Real stepping-forward was larger than for recurrent-fallers and fallers. Nonetheless, it was the Estimated stepping-forward variable, which best explained the risk of falls, either of falling recurrently or of falling. This variable also showed higher results for non-fallers compared to recurrent-fallers and fallers and evidenced to be strongly related to the Real stepping-forward for general participants. This relationship is in line with Konczak and colleagues’ [28] observations that also found a link between older adults’ perception and their action capabilities, although regarding stair climbing. Thus, the present study results indicate that, despite the Real *minus* Estimated bias, most participants have a good perception of their ability to step-forward, suggesting that an appropriated approach explaining the occurrence of falls requires the combination of the physical ability to perform the task (Real stepping-forward) and the respective perception judgment (Estimated stepping-forward). This hypothesis is in accordance with Fajen and colleagues [29], who argued that secure behaviors in daily routine require the ability to perceive which actions are possible within the limits of capabilities.

Although some results from the present study are in line with Fajen and colleagues’ [29] suggestion that the judgment accuracy of the Estimated and Real stepping-forward could avoid falls occurrence, the present study showed that a large underestimation of the real capabilities—Absolute-error—is inversely associated with falls occurrence. In contrast, the alternative built model showed that the higher the overestimated Absolute-error, the greater the risk of falls. These results evidenced that the underestimation bias works as a safety buffer to avoid falls, and that over excessive belief on the stepping-forward task performance (evidenced by an overestimated bias) works as a risk for falls occurrence. Thus, concerning the ecological approach of falls and *affordances* [6,30], falls may result from a failed action consequent from an overestimation mismatch between what older adults believe they can do and what in reality, they are capable. Such mismatch can put older adults at risk by leading them to perform actions that they are no longer physically capable of doing.

The use of the equations generated by multivariate binary logistic regression modeling evidenced that a larger underestimated Absolute-error may partially compensate a smaller Estimated stepping-forward as regards to the risk (probability) of falling recurrently and of falling and vice versa (see Fig 1). Nonetheless, ROC analysis allowed the establishment of cut-off values on these variables, which are red-flags for these negative occurrences and should be used as a reference to compare with the values achieved by each subject on the SF-APT. For instance, an older adult who estimates to perform a step-forward of 50 cm and who performs the task with an underestimated Absolute-error of 2 cm (results corresponding to the 25^th^ percentile, see Table 1) is at a very high risk of falling recurrently and of falling since he/she is categorized either as a recurrent-faller as well as a faller (see Fig 1). However, the comparison of his/her results with the alert red-flag gives the information that if the older adult increases his/her Estimated stepping-forward by ≥8 cm [red-flag for falling recurrently (58 cm)—gets Estimated stepping-forward (50 cm)] = 8 cm], and increases his/her underestimated Absolute-error on ≥ 3 cm [red-flag for falling recurrently (5 cm)—gets Estimated stepping-forward (2 cm) = 3 cm], this person would no longer be a recurrent-faller. Moreover, if the older adult increases his/her Estimated stepping-forward on ≥12 cm [red-flag for falling (62 cm)—gets Estimated stepping-forward (50 cm) = 12 cm] and increase his/her underestimated Absolute-error on ≥4 cm [red-flag for falling (6 cm)—gets Estimated stepping-forward (2 cm) = 4 cm], this person would no longer be a faller.

The present study was able to identify new modifiable key outcomes as risk factors for falls. Such finding is particularly important considering recent studies [31] alerting that, to be effective, falls prevention interventions must target modifiable risk factors. Although the development of older adults’ self-confidence may be important for the maintenance of physical functioning and, therefore, contribute to falls prevention [32], the present study suggests that excessive belief in performance ability is a serious risk to falls occurrence. Thus, in what concerns to falls prevention, it would be crucial to increase individuals’ stepping-forward capacity and individuals’ perception of action boundaries for a securer stepping-forward estimation, that is, ensuring that the Real-Estimation bias (Error-tendency) would be of underestimation.

The present study findings suggest that SF-APT should be included in falls risk screening once a year, as recommended by the American Geriatric Society and the Centers for Disease Control and Prevention [33] because it has shown to assess a key risk factor for falls and most likely, susceptible to change. The established risk cut-off will allow quantifying how far the assessed older adult is of the safe test result and, therefore, the definition of effective rehabilitation programs designed to improve the impaired high fall risk factor. Nonetheless, future researches shall study the role of affordance perception outcomes on falling risk considering other risk factors, such as the previous falls, gait, and balance reported by Palumbo et al. [5], to include the SF-APT in the algorithms recommended for falls risk screening.

Limitations of this study include the use of a retrospective recall for assessing falls occurred in the previous 12 months. Nonetheless, this data was considered acceptable given that a healthy cognitive function was required for all participants, and that the information was collected by detailed interviews focusing on the circumstances surrounding each fall. Still, it is possible that there was an underestimation of the accessed falls. Although gender was not computed as significantly explaining fall occurrence in multivariate modeling, the relatively small number of men compared to women may be a limitation of the study since some authors point to gender as a moderator of fall occurrence [34]. Another limitation is the relatively small number of fallers, and the small number of recurrent-fallers, limiting the present study’s statistical power. Therefore, more extensive research could be necessary to confirm and generalize the findings of this study to other work sites. Nonetheless, the predicted probabilities generated by multivariate binary regression modeling (concerning falling and falling recurrently), and the predicted probabilities produced by the cross-validation procedures (concerning falling and falling recurrently) were both significant. Finally, as the relationship between SF-APT variables and reported falls was explored cross-sectionally, some precautions should be taken in the interpretation of directionality and cause-effect relationships. Hence, future follow-up researches using the SF-APT and prospective falls assessment should be carried out with older populations. Besides these limitations, the SF-APT is quick and easy to apply, is well tolerated by older adults, requires ordinary material and can be used in several settings by professionals of different fields, as a geriatric screening for determining the risk of falling.

## Conclusion

The SF-APT Estimated stepping-forward and the Absolute-error in interaction with Error-tendency were confirmed as the key outcomes which should be used to assess the risk of falls of community-dwelling older adults. A qualitative and quantitative relationship was found between these outcomes and the risk of falls. Moreover, it was found that estimating a boundary stepping-forward larger than 58 cm plus showing an underestimation bias larger than 5 cm (values above 42^nd^ percentile) avoids older adults to be recurrent-fallers while estimating a boundary stepping-forward larger than 62 cm plus showing an underestimation bias larger than 6 cm (values above 54^th^ percentile) avoids older adults to be fallers. Giving the relevance of these results, interventions targeting the prevention of falls should consider the above key outcomes and the respective cut-offs as alert red-flags. Therefore, SF-APT would be a valuable tool for falls risk assessment in community-dwelling older adults.

## Supporting information

S1 Fig Probability falling and of falling recurrently associated with the key SF-APT outcomes (estimated stepping-forward, and the Absolute-error in interaction with Error-tendency) and respective cut-offs discriminating fallers from non-fallers or recurrent-fallers from non-fallers. (DOCX)

S1 Table Description of participants SF-APT outcomes data. (DOCX)

S2 Table The two most parsimonious and fit build binary logistic regression models and respective key SF-APT variables explaining falling and falling recurrently (falling vs. non-falling model: N = 347; falling recurrently vs. non-falling model: N = 263). (DOCX)
