# Supplementary material for: Stepping-forward affordance perception test cut-offs: Red-flags to identify community-dwelling older adults at high risk of falling and of recurrent falling
Source: PLoS One. 2020 Oct 8;15(10):e0239837. doi: 10.1371/journal.pone.0239837 (PMC7544084; doi:10.1371/journal.pone.0239837)
Supplement: S1 Fig — (DOCX) [file pone.0239837.s001.docx]

|  |  |
| --- | --- |

**Fig 1. Probability falling and of falling recurrently associated with the key SF-APT outcomes (Estimated stepping-forward, and the Absolute-error in interaction with Error-tendency) and respective cut-offs discriminating fallers from non-fallers or recurrent-fallers from non-fallers.**
