# Supplementary material for: Stepping-forward affordance perception test cut-offs: Red-flags to identify community-dwelling older adults at high risk of falling and of recurrent falling
Source: PLoS One. 2020 Oct 8;15(10):e0239837. doi: 10.1371/journal.pone.0239837 (PMC7544084; doi:10.1371/journal.pone.0239837)
Supplement: S1 Table — (DOCX) [file pone.0239837.s002.docx]

**Table 1. Description of participants SF-APT outcomes data.**

|  | **Mean (95%CI) or prevalence** | | | | **P5** | **P10** | **P25** | **P50** | **P75** | **P90** | **P95** |
| --- | --- | --- | --- | --- | --- | --- | --- | --- | --- | --- | --- |
|  | **Non-fallers** | **Fallers** | **Recurrent-fallers** | **Total** |  |  |  |  |  |  |  |
| Estimated stepping-forward (cm) | 63.7 (61.6-65.9) | 57.1 (54.7-59.5) | 55.4 (52.0-58.7) | 60.9 (59.3-62.6) | 36 | 41 | 50 | 61 | 71 | 80 | 88 |
| Real stepping-forward (cm) | 70.7 (68.6-72.7) | 61.7 (59.4-64.1) | 58.6 (55.3-61.9) | 66.9 (65.3-68.5) | 41 | 45 | 58 | 67 | 76 | 85 | 94 |
| Algebraic-error^a^ (cm) | 7.0 (5.7-8.2) | 4.6 (3.4-5.9) | 3.3 (1.3-5.3) | 6.0 (5.1-6.9) | -6 | -3 | 1 | 5 | 10 | 18 | 22 |
| Absolute-error (cm) | 8.4 (7.4-9.5) | 6.7 (5.7-7.7) | 6.5 (5.2-7.9) | 7.7 (7.0-8.4) | 0 | 1 | 2 | 6 | 11 | 18 | 22 |
| Absolute-percent-error (%) | 11.9 (10.4-13.3) | 11.0 (9.5-12.5) | 11.3 (9.0-13.5) | 11.5 (10.5-12.5) | 0.4 | 1.5 | 3.3 | 9.1 | 16.7 | 25.8 | 33.3 |
| Error-tendency (%) |  |  |  |  |  |  |  |  |  |  |  |
| Overestimation | 17.9 | 29.5 | 33.9 | 22.8 |  | -- | -- | -- | -- | -- | -- |
| Underestimation | 82.1 | 70.5 | 66.1 | 77.2 |  | -- | -- | -- | -- | -- | -- |

^a^Real-Estimated

Data are Mean and 95% Confidence Interval (CI) or Prevalence. and Percentile Values (P).
