# Supplementary material for: Stepping-forward affordance perception test cut-offs: Red-flags to identify community-dwelling older adults at high risk of falling and of recurrent falling
Source: PLoS One. 2020 Oct 8;15(10):e0239837. doi: 10.1371/journal.pone.0239837 (PMC7544084; doi:10.1371/journal.pone.0239837)
Supplement: S2 Table — (DOCX) [file pone.0239837.s003.docx]

**Table 2. The two most parsimonious and fit build binary logistic regression models and respective key SF-APT variables explaining falling and falling recurrently (Falling vs. Non-falling Model: N=347; Falling recurrently vs. Non-falling Model: N = 263).**

| **Model** | **Key variables** | **OR (95%CI)** | **Optimal**  **Cut-off point** | **Specificity (%)** | **Sensitivity (%)** | **AUC (95%CI)** |
| --- | --- | --- | --- | --- | --- | --- |
| Falling | Estimated stepping-forward (cm) | 0.964 (0.948-0.979) | 0.412 | 0.587 | 0.671 | 0.665 (0.608-0.723) |
|  | Absolute-error (cm) * Error-tendency ^a^ |  |  |  |  |  |
|  | Overestimation (0) |  |  |  |  |  |
|  | Underestimation (1) | 0.941 (0.910-0.973) |  |  |  |  |
| Falling recurrently | Estimated stepping-forward (cm) | 0.951 (0.931-0.973) | 0.261 | 0.716 | 0.661 | 0.728 (0.655-0.797) |
|  | Absolute-error (cm) * Error-tendency |  |  |  |  |  |
|  | Overestimation (0) |  |  |  |  |  |
|  | Underestimation (1) | 0.914 (0.868-0.962) |  |  |  |  |

*Interaction between variables

Data are Multivariate Odds Ratio (OR) and 95% Confidence Interval (CI), Cut-off points for π, Specificity, Sensibility, and Area Under the ROC Curve (AUC) and 95% CI.
